# Factors Within the Endoneurial Microenvironment Act to Suppress Tumorigenesis of MPNST

**DOI:** 10.3389/fncel.2018.00356

**Published:** 2018-10-11

**Authors:** Jo Anne Stratton, Peggy Assinck, Sarthak Sinha, Ranjan Kumar, Aaron Moulson, Natalya Patrick, Eko Raharjo, Jennifer A. Chan, Rajiv Midha, Wolfram Tetzlaff, Jeff Biernaskie

**Affiliations:** ^1^Hotchkiss Brain Institute, University of Calgary, Calgary, AB, Canada; ^2^Department of Comparative Biology and Experimental Medicine, Faculty of Veterinary Medicine, University of Calgary, Calgary, AB, Canada; ^3^Alberta Children’s Hospital Research Institute, University of Calgary, Calgary, AB, Canada; ^4^Department of International Collaboration on Repair Discoveries, The University of British Columbia, Vancouver, BC, Canada; ^5^Graduate Program in Neuroscience, The University of British Columbia, Vancouver, BC, Canada; ^6^Arnie Charbonneau Cancer Institute, University of Calgary, Calgary, AB, Canada; ^7^Department of Pathology and Laboratory Medicine, University of Calgary, Calgary, AB, Canada; ^8^Department of Clinical Neurosciences, Cumming School of Medicine, University of Calgary, Calgary, AB, Canada

**Keywords:** peripheral nerve, sarcoma, Schwann cell, MPNST, CNTF, endoneurium, epineurium, microenvironment

## Abstract

**Background:** Deciphering avenues to adequately control malignancies in the peripheral nerve will reduce the need for current, largely-ineffective, standards of care which includes the use of invasive, nerve-damaging, resection surgery. By avoiding the need for en bloc resection surgery, the likelihood of retained function or efficient nerve regeneration following the control of tumor growth is greater, which has several implications for long-term health and well-being of cancer survivors. Nerve tumors can arise as malignant peripheral nerve sheath tumors (MPNST) that result in a highly-aggressive form of soft tissue sarcoma. Although the precise cause of MPNST remains unknown, studies suggest that dysregulation of Schwann cells, mediated by the microenvironment, plays a key role in tumor progression. This study aimed to further characterize the role of local microenvironment on tumor progression, with an emphasis on identifying factors within tumor suppressive environments that have potential for therapeutic application.

**Methods:** We created GFP-tagged adult induced tumorigenic Schwann cell lines (iSCs) and transplanted them into various *in vivo* microenvironments. We used immunohistochemistry to document the response of iSCs and performed proteomics analysis to identify local factors that might modulate divergent iSC behaviors.

**Results:** Following transplant into the skin, spinal cord or epineurial compartment of the nerve, iSCs formed tumors closely resembling MPNST. In contrast, transplantation into the endoneurial compartment of the nerve significantly suppressed iSC proliferation. Proteomics analysis revealed a battery of factors enriched within the endoneurial compartment, of which one growth factor of interest, ciliary neurotrophic factor (CNTF) was capable of preventing iSCs proliferation *in vitro*.

**Conclusions:** This dataset describes a novel approach for identifying biologically relevant therapeutic targets, such as CNTF, and highlights the complex relationship that tumor cells have with their local microenvironment. This study has significant implications for the development of future therapeutic strategies to fight MPNSTs, and, consequently, improve peripheral nerve regeneration and nerve function.

## Importance of Study

These studies provide several major findings. First, we develop a reproducible *in vitro* model to study MPNST from isolated adult rodent Schwann cells (termed iSCs) that following transplantation, share striking phenotypic resemblance to human MPNST tumors. Second, our results underscore the importance of tissue microenvironment in promoting tumorigenic growth and identify the endoneurial compartment within the peripheral nerve as a unique microenvironment enriched in tumor suppressive factors. Third, by probing uniquely expressed proteins within the endoneurial compartment, we demonstrated an autonomous role for CNTF to block proliferation of iSCs mimicking the inhibition observed when grafted iSCs are contained within the endoneurial compartment *in vivo*. Together, these experiments provide evidence for a tumor suppressive endoneurial niche that functions to repress proliferation and identifies CNTF signaling as a potential therapeutic avenue for MPNST.

## Introduction

Malignancies within the peripheral nerve usually arise from aberrant Schwann cell proliferation (Miller et al., 2006; Chen et al., 2014) giving rise to malignant peripheral nerve sheath tumors (MPNST), a highly aggressive and largely untreatable form of soft tissue sarcoma, often culminating in patient death within ten years(LaFemina et al., 2013). MPNSTs occur in association with inherited syndromes, such as Neurofibromatosis Type 1 (NF1) but can also occur sporadically (46–48% of cases) (LaFemina et al., 2013). Although numerous treatment modalities have been assessed for treating MPNSTs (i.e., radiation therapy, surgical resection) they show moderate to poor results long term (LaFemina et al., 2013).

Deciphering avenues to adequately control malignancies in the peripheral nerve will potentially improve the current standard of care. More effective adjunctive drug therapy may allow modification of en block procedures, toward a nerve-sparing approach, to permit function to be retained.

There is strong evidence to suggest that distinct locations within the peripheral nervous system are prone to tumor formation and progression. For example, several studies have reported the capacity of nerve sheath tumors to form, with increased tumor burden, within the proximal nerves and plexus compared to the distal nerves (Plotkin et al., 2012) and are increasingly metastatic in the intrathoracic and dorsal root ganglion (DRG) regions compared to subdiaphragmatic regions (Kourea et al., 1998). One potential difference between these anatomical regions is the presence of distinct local microenvironments unique to each location (Le et al., 2010; Lavasani et al., 2013). Moreover, transplanting NF1-mutant cells into several distinct locations within healthy rodents revealed that aberrant cell growth was especially pronounced when introduced into distinct regions (Le et al., 2010). The factors responsible for these divergent effects remain to be elucidated.

In line with this, others have observed similar phenomena following injury (Ribeiro et al., 2013; Schulz et al., 2016). Recently, Ribeiro and his colleagues found that when NF1 mutation was induced in adult Schwann cells, tumors formed in the nerve, but intriguingly *only* post-injury, and were restricted *only* to the injury site. In this context, the authors concluded that tumor formation must involve an interplay between Schwann cell-associated NF1 mutation and injury environments. They suggested that although the nerve is generally a “tumor suppressive environment,” an insult can locally alter the composition and concentration of factors present at the injury site, consequently allowing unregulated cell growth. Indeed, cytokine-releasing mast cells at the injury site have been shown to play a role in nerve tumor progression by introducing factors that are not typically present within the intact nerve (Yang et al., 2008). Importantly, the authors also noted that tumors did not form distal to the injury site – an area of the nerve that undergoes Wallerian degeneration post-injury. Since this area is subjected to similar injury cues, including the presence of mast cells (Gaudet et al., 2011), such findings suggest that injury-associated factors are not the sole mediators of tumorigenicity in this context.

Another plausible explanation for the formation of tumors at the injury site is the breakdown of connective tissue barriers as a result of the mechanical force exerted at the injury site itself (Olsson and Kristensson, 1973). The perineurial barrier, a thin layer of perineurial cells and collagen (Riccardi, 2007), acts as a specialized blood-nerve barrier in health (similar to that of the central nervous systems blood-brain barrier) (Allt and Lawrenson, 2000), but becomes compromised at sites of nerve injury (Haftek and Thomas, 1968). In homeostatic conditions, this specialized perineurial barrier with tight junctions prevents components from the endoneurium, where axons and Schwann cells reside, to diffuse freely into the epineurium, where large amounts of connective tissue resides (Olsson and Kristensson, 1973), as well as vice versa. Importantly, long term compromised perineurial barrier function post-injury is spatially confined *only* to the site of injury and does not extend distally (Olsson and Kristensson, 1973).

The potential contribution of a compromised barrier function to tumor progression becomes especially plausible when the types of cells and factors present within each defined compartment are considered. The epineurium harbors fibroblasts, adipocytes, endothelial cells, blood vessels, mast cells and large amounts of collagen (Norris et al., 1985; Verheijen et al., 2003). On the other hand, the endoneurial compartment mainly harbors Schwann cells (90%) and axons as well as a small number of neural-crest derived fibroblasts, endothelial cells, immune cells and small amounts of collagen (Sunderland, 1945; Riccardi, 2007; Weiss et al., 2016). Interestingly, several studies have shown that factors/cells known to be present within the epineurium enhance tumor progression (Fang et al., 2014; Kuzet and Gaggioli, 2016; McDonald et al., 2016), while several factors known to be present within the endoneurial compartment suppress Schwann cell proliferation (Parrinello et al., 2008). As such, one might hypothesize that Schwann cell-derived tumorigenesis is a consequence of perineurium breakdown and consequent exposure of latent tumorigenic cells to factors previously restricted to the epineurial compartment competing with suppressive factors within the endoneurium.

To elucidate the role of microenvironment on tumor progression, we subjected healthy adult Schwann cells to a mutagenic environment *in vitro* following which they were transplanted into various *in vivo* anatomical microenvironments. Following transplantation, mutagenic Schwann cells share striking phenotypic resemblance to human MPNST tumors. Intriguingly, when these same mutagenic induced tumor Schwann cells (iSCs) are injected and contained within the endoneurial compartment, they exit cell cycle and down-regulate tumor markers. Using quantitative proteomics to screen for factors that were uniquely present within the endoneurial compartment, we identified ciliary neurotrophic factor (CNTF), a growth factor known for promoting Schwann cell differentiation (Reynolds and Woolf, 1993), as a factor that was not only enriched within the endoneurium, but exhibited an autonomous capacity to inhibit proliferation of mutagenic Schwann cells. Uncovering the signals in the endoneurial niche that bias Schwann cells against hyperproliferation can safeguard against nerve malignancies and pave the path for regeneration and return of nerve function for cancer survivors.

## Materials and Methods

### *In vitro* Induction of Tumor-Forming Adult Schwann Cells (iSCs)

Sciatic nerves were collected from three adult Sprague Dawley rats, then Schwann cells were isolated using a modified protocol from previously described methods (Mirfeizi et al., 2017). Briefly, nerves were dissected and minced, and subsequently incubated in collagenase Type IV (Worthington) at 37°C for 40 min. Samples were then centrifuged (1000 rpm for 5 min), then re-suspended in Schwann cell media containing DMEM and F12 (3:1), neuregulin-1 type III (50 ng/ml), forskolin (5 μM), B27 supplement (1%), and penicillin (100 μg/ml)/streptomycin (100 units/ml). Cells were plated on poly-D-Lysine (20 μg/ml) and laminin (4 μg/ml) coated surfaces (BD Bioscience) and cultured at 37°C in a 5% CO_2_ incubator. Cultures were also supplemented with plasmocin (25 μg/mL, Invitrogen) and fungizone (40 ng/ml, Invitrogen) for the first month of culturing to prevent mycoplasma and fungal growth. Triple-filtered fetal bovine serum (1–5% FBS; Hyclone) was added for the first 5 days of culturing. Cells were maintained *in vitro* for 3–4 months. For passaging, media was removed and TrypLE-express (Invitrogen) was added to cultures and incubated at 37°C for 5 min. All detached cells were collected and plated at a density of 50,000 cells/ml for further maintenance. Tumor-forming characteristics of adult Schwann cells were induced by extended growth and repeated passage *in vitro* as previously described (Funk et al., 2007). Spherical colony formation (loss of contact mediated growth inhibition) and growth factor independence was observed by 3 months in culture. Cells were fed every 4th day and treated identically.

### Lenti-Viral Labeling

To track cells following *in vivo* transplantation, cells were transduced with a GFP-expressing lenti-viral vector as previously described (Mirfeizi et al., 2017). All lentivirus work was undertaken in a biohazard level 2+ laboratory using previously established protocols. Cells (70% confluence) were incubated overnight in DMEM containing GFP-expressing lentiviral particles and polybrene (8 μg/ml). The next day, fresh media was applied and cells were left to grow to 100% confluence. Cells were then dissociated and FACs sorted (BD FACS Aria III) to further purify the GFP+ cell fraction.

### Karyotyping

To assess chromosomal integrity, karyotyping was performed on iSCs using previously described methods (Fan et al., 2014). Cultures (70% confluence) were treated with KaryoMAX Colcemid solution (30 ng/mL, Gibco) for 4 h at 37°C. Schwann cells were then detached from dishes, centrifuged, then re-suspended in cell hypotonic solution (CHS; 40 mM Kcl, 20 mM HEPES, 0.5 mM EGTA and 9 mM NaOH) and incubated for 1 h at 37°C. After 1 h, the mix was fixed with acetic acid/methanol (1:3) and G-banding analysis was performed in the Clinical Genetics Facility at the Alberta Children’s Hospital.

### *In vitro* Treatments, Immunocytochemistry and Imaging

To assess the response of iSCs to different environmental stimuli, iSCs were plated onto 96-well plates at 10,000 cells/mL. Cells were treated with base media in the presence of neuregulin (100 ng/ml, R&D), CNTF (0–100 ng/ml, Peprotech), or media pre-conditioned with epineurial tissue, endoneurial tissue or spinal cord tissue. To prepare media for conditioned media experiments, equal wet weights of healthy epineurial tissue or endoneurial tissue from sciatic nerves, or white matter from thoracic spinal cords was dissected from C57BL/6 mice (*n* = 8–10), then maintained in DMEM and F12 (3:1), B27 supplement (1%), penicillin (100 μg/ml)/streptomycin (100 units/ml) and 10% FBS for 4–6 days before filtering (0.4 μm), and snap freezing at −80°C until iSCs 96-well experiments were ready. Depleted media alone controls were treated identically except no explants were added. Three days post-treatment, cells were washed with sterile PBS and then fixed in 4% paraformaldehyde (PFA) for 5–10 min. For immunocytochemistry, cells were permeabilized using 0.5% triton X-100 and 5% BSA for 1–2 h at room temperature. Primary antibodies (rat anti-Ki67 (clone SolA15, 14569882, eBiosciences), mouse anti-nestin (SC23927, Santa Cruz) were incubated overnight (1:200) at room temperature. Unbound primary antibodies were washed and cells were subsequently incubated with Alexa-conjugated secondary antibodies (1:200, Invitrogen) at room temperature for 1–2 h, washed, and nuclei were counterstained with Hoechst (1:1000, Sigma) and imaged using ImageXpress (Molecular Devices). The sum of cells (Ki67+ NES+ Hoechst+ versus NES+ Hoechst+) in 12 images (20×) per well was obtained, then normalized to obtain a percentage value for each well. This value was subsequently averaged across three replicates per condition. Experiments were replicated on 3 independent days.

### Animal Care and Surgery

Animal procedures were approved by the University of Calgary and University of British Columbia Animal Care Committees in compliance with the Guidelines of the Canadian Council of Animal Care. To assess the impact of *in vivo* microenvironments on the capacity of iSCs to form tumors, adult 8–12 weeks immune-deficient Foxn1^*nu*^, NOD/CB17-*Prkdc^scid^*/NcrCrl mice (for nerve and skin injections), or Sprague Dawley rats (for spinal cord injections) were used [Charles River Laboratories (Senneville, QC, Canada)]. Rats were treated with Cyclosporin A and GM1 natural killer cell antibodies for immunosuppression. Rodents were maintained on a 12-h light cycle in a temperature-controlled environment with unlimited food and water. For cell transplants, rodents were anesthetized using isofluorane (5% induction and 2% maintenance) and given subcutaneous injections of 0.1 mL (0.03 mg/mL) buprenorphine for pain relief. For nerve and spinal cord transplants, an injury was first induced prior to transplants in order to create an environment permissive for tumor formation (Ribeiro et al., 2013). Surgery sites were sterilized and then the sciatic nerve (including endoneurial and epineurial compartments) or spinal cords were exposed and crush injured using #5 forceps (nerve) or an Infinite Horizon Impactor, 200 KD (spinal cord). Immediately after (nerve) or 2 weeks after (spinal cord) injury, each distal nerve received a 2 μl volume injection (100,000 cells) and each spinal cord received a 5 μl volume injection (500,000 cells) of GFP+ve nerve or skin-derived iSCs (*n* = 3–4 rodent per donor, 2–3 donors) using a 33-gauge Hamilton syringe. iSCs were suspended in neuregulin (500 ng/ml) and fast green (1%) in DMEM at a density of 50,000 cells/μl. Importantly, this injection strategy resulted in the presence of iSCs in the endoneurial (tibial fascicles) and epineurial (surrounding the fascicles) compartments. For skin transplants, GFP transduced iSCs were injected intradermally into the back skin of NOD/CB17-*Prkdc^scid^*/NcrCrl mice. In all cases, tissue was collect within 2–4 months of surgeries.

### Tissue Processing, Immunohistochemistry and Imaging

Briefly, nerve, skin, and spinal cord tissue was harvested at 2–4 months post-transplant, then fixed overnight with 4% PFA. For spinal cords, 4% PFA cardiac perfusion was also performed. Tissue was then left in 30% sucrose overnight, then frozen in OCT (VWR International) and stored at −80°C. For comparisons to MPNST, archived human and rodent samples were fixed overnight in 10% neutral buffered formalin, processed through graded ethanol’s and xylene, and embedded in paraffin. Sections were cut at 4 μm thickness and stained with hematoxylin and eosin (H&E). For IHC, heat-induced antigen retrieval was performed in 10 mM sodium citrate for 25 min in microwave prior to staining with the following primary antibodies: S100 (DAKO) rabbit polyclonal 1:3,000 dilution and Desmin (DAKO) mouse monoclonal D33 1:400 dilution. Detection of staining was performed using Envision+ DAB kit (DAKO) per manufacturer’s protocol with hematoxylin as a counterstain. Archival material from MPNST surgical samples were obtained from the Clark Smith Tumor Bank at the University of Calgary (in collaboration with Calgary Laboratory Services) with ethics approval from the Health Research Ethics Board of Alberta – Cancer Committee. For immmunohistochemitry for PFA fixed tissue, sections were cut using a Leica cryotome at 10–20 μm. Sections were then permeabilized with 0.5% triton-X 100 and blocked with 5% BSA or 10% NDS. Primary antibodies were incubated overnight (rabbit anti-Ncad, AB12221 Abcam; rat anti-Ki67, 14569882, eBiosciences; goat anti-CNTF, AB557 R&D; sheep anti-ErbB3, AF4518 R&D), washed with PBS, and then Alexa-conjugated secondary antibodies (1:200, Invitrogen) were applied for 2 h at room temperature. Hoechst was used to stain nuclei (1:1000, Sigma) and mounted with Permafluor (Thermo Fisher Scientific). Image collection and quantification was done using a Leica SP8 confocal microscope. Two images per condition at 4 mm distal to the injury site was collected using a 63× objective lens and Z-stack (eight planes) features. Images (maximum projection) were analyzed using ImageJ (NIH). These counts were then expressed as a ratio (i.e., GFP+ve Ki67+ve cells/GFP+ve cells) and averaged within each animal (*n* = 5 animals/group).

### Proteomics

In order to assess the protein composition of the fascicular portion of the nerve, sciatic nerves were firstly extracted from healthy adult C57BL/6 mice (*n* = 8). Using fine forceps the outer sheaths (ie. epineurium and perineurium) of the sciatic nerves were carefully removed. At this stage the contents of the endoneurium (individual myelinated axons) were clearly distinguishable, and were minced and incubated before processing for protein collection. Equal wets weights of the dorsal column from the same mice were also collected as a comparison. Protein was extracted with SDS gel sample buffer [0.2 M Tris, 5 mM EDTA, 1 M Sucrose, SDS and dithiothreitol (DTT)]. Protein was then run on 10% acrylamide gel containing SDS to separate out proteins that were most likely of interest (10–60 kDa).

Gel plugs were washed in 50 mM ammonium bicarbonate/acetonitrile (50:50, v/v) then incubated in 100% acetonitrile. After being air dried, proteins were reduced with DTT (10 mM) at 56°C and alkylated with iodoacetamide (50 mM) at 24°C. Gel plugs were washed then rehydrated with a trypsin solution (Promega; 0.02 μg/ul, 10% acetonitrile) for 2 h at 0°C then placed in buffer for 16 h at 37°C. Supernatant (tryptic peptides) was transferred to acidifying solution [acetonitrile/water/10% trifluoroacetic acid (60:30:10, v/v)]. Gel plugs were washed with the same acidifying solution and combined. Samples were then lyophilized and resuspended in 1% formic acid. The tryptic peptides were analyzed by liquid chromatography (LC; Agilent 1260 Infinity chip cube interface) tandem mass spectrometry (MS/MS) on an Agilent 6550 iFunnel quadrupole (Q)- time-of-flight (TOF) mass spectrometer. The LC and the Q-TOF were both controlled by MassHunter (B.05.00). The capillary pump used: A1 (97% water, 2.9% acetonitrile, 0.1% formic acid) and B1 (90% acetonitrile, 9.9% water; 0.1% formic acid) solutions; and, the nanopump used: A1 (97% water, 2.9% acetonitrile, 0.1% formic acid) and B1 (97% acetonitrile, 2.9% water; 0.1% formic acid) solutions. Tryptic peptides (1 μl) were loaded onto a C18 trap column of an Agilent chip operating in enrichment mode using the capillarity pump of the LC system at a flow rate of 2.5 μl/min. Elution of the peptides was performed using a 25 min linear gradient from 3% to 50% B1 generated by the nanopump operated at 0.3 μl/min. The peptides were electrosprayed into the Q-TOF using an ionization voltage of 1950V and a 275°C heated drying gas at a flow of 13 l/min. The Q-TOF was operated in positive auto MS/MS mode. The precursor ions with a m/z comprised between 275 and 1700 were acquired at a scan rate of 250 ms/spectrum and the 10 most abundant precursors for each cycle having a charge higher than 1, an intensity of at least 1000 counts and a peptidic isotopic model were fragmented by collision induced dissociation. Fragment ions having a m/z comprised between 50 and 1700 were acquired at a scan rate of 333.3 ms/spectrum. The collision energy was calculated for each precursor based on its charge and its mass. An active exclusion which was released after 1 spectrum and 0.2 min was applied to avoid re-acquiring the same precursor.

For data extraction, a peptidic isotopic model with a maximum charge state of 6 was used. The peak filtering for MS/MS data was set up at an absolute height of at least 10 counts and relative height of the most abundant peak of at least 0.1%. Using Mascot algorithm (2.4), NCBI database search was performed. Parameters included: trypsin as enzyme, maximum number of missed cleavage of 1, a peptide charge of 2+, 3+, and 4+, cysteine carbamidomethylation as fixed modification, methionine oxidation as variable modification and a mass error tolerance of 20 ppm. A mass error tolerance of 0.2 Da was selected for the fragment ions. Only peptides identified with a score having a confidence higher than 95% were kept for further analysis.

### Statistical Analysis

All statistical analysis was performed using GraphPad Prism (v5.0). Statistical comparisons were performed using a two-tailed unpaired Student’s *t*-test or One-way ANOVA followed by Tukey’s *posthoc* test. A *p*-value of <0.05 was considered statistically significant. All graphs are presented as mean ± standard error of mean (SEM).

## Results

### The Development of a Model for MPNST

**FIGURE 1:**
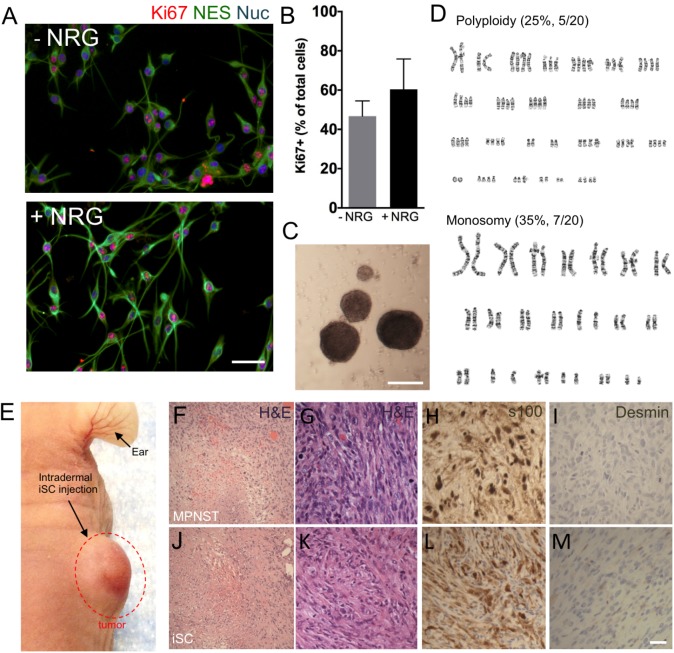
The development of a malignant peripheral nerve sheath tumors (MPNST) model. **(A)** Representative immunocytochemical images of induced tumor Schwann cells (iSCs) either deprived of growth factors (−NRG) or treated with neuregulin and forskolin (+NRG, 50 ng/ml neuregulin, 5 mM Forskolin). Note the similar numbers of Nestin+ (green, NES), Hoechst+ (blue) iSCs that express Ki67 (red) across both conditions. **(B)** Quantification of percentage of Ki67^+^ cells revealed no difference between groups (Student’s *t*-test, *n* = 4, *P* = 0.3). **(C)** Note the presence of sphere formation when iSCs are grown in growth factor deprived conditions. **(D)** Karyotyping analysis indicates multiple chromosomal abnormalities, including polyploidy and monosomy cells. **(E)** Intradermal iSC injections into back skin resulted in the development of tumors within 16 weeks. **(F–M)** Representative histological images of H&E **(F,G,J,K)**, s100 **(H,L)** and Desmin **(I,M)** from a biopsied sample of human MPNSTs **(F–I)** and from a sample from iSCs **(J–M)**. Note the presence of necrosis, mitosis and spindle-shaped cells in both samples **(F,G** and **J,K)**. Also note the presence of S100 **(H,L)** immunoreactivity, as well as the lack of Desmin immunoreactivity **(I,M)** in both samples. Scale bars = **A** (50 μm), **C** (200 μm), **G–I** (20 μm).

To develop an MPNST model, we isolated primary Schwann cells from adult uninjured nerves (*n* = 3) and subjected these cells to identical mutagenic *in vitro* conditions as described by Funk and colleagues rodent experiments (Funk et al., 2007). Following 3–4 months of *in vitro* processing, adult Schwann cells consistently demonstrated features typical to tumor cell lines, including growth factor independent growth, loss of contact-mediated growth suppression, and abnormal chromosomes (**Figure 1**). Immunocytochemical assessment of proliferation kinetics indicated that iSCs were capable of proliferating at similar rates even in the absence of growth factors (neuregulin and forskolin; **Figures 1A,B**); indicating a loss of growth factor-dependent growth (Funk et al., 2007). Subsequent quantification revealed that the percentage of Ki67+ iSCs did not statistically differ between iSCs cultured in the presence or absence of growth factors critical to SC survival (Student’s *t*-test, *n* = 4, *p* = 0.3). Also, iSCs formed non-adherent spherical colonies indicating a loss of contact-mediated cell cycle arrest—another common feature of uncontrolled proliferation (**Figure 1C**). Finally, we also demonstrated that there were several chromosomal abnormalities, including polyploidy and monosomy present within all iSC cultures (**Figure 1D**). Together, such findings indicate that Schwann cells exposed to extended *in vitro* cell culture exhibit reproducible transformation of otherwise healthy Schwann cells.

In order to determine the subtype of tumors generated by iSCs, we assessed several *in vivo* features of iSC-generated tumors following transplantation into the skin and compared these to human MPNST tumor biopsies collected at the Clark Smith Tumor Bank at the University of Calgary. Following subcutaneous injection of iSCs, 10/12 (83%) of cases showed a dermal growth at the injection site (**Figure 1E**). Consistent with MPNST (**Figure 1F–I**), iSC-generated tumors consisted of solid sheets and fascicles comprising elongated spindled cells with enlarged atypical oval and tapered nuclei, and moderate amounts of cytoplasm (**Figures 1J,K**). Tumor cells were mitotically active and displayed foci of intratumoral necrosis. Immunohistochemical staining showed that the tumor cells were focally immunoreactive for S100 (**Figure 1L**) and were negative for desmin (**Figure 1M**). Together, the histologic and immunophenotypic findings of iSC-generated tumors recapitulate those of human MPNST.

### Endoneurial Microenvironment Suppresses Tumorigenesis of MPNST

**FIGURE 2:**
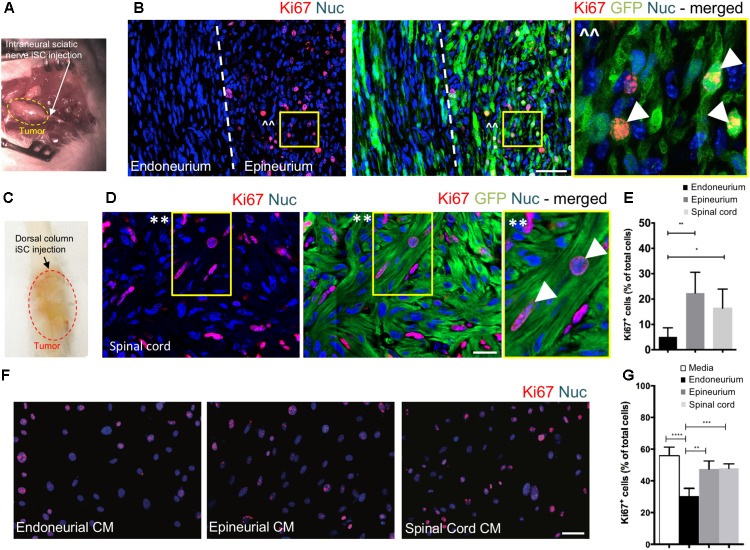
The endoneurial microenvironment suppresses tumorigenesis of MPNST. **(A)** Intraneural iSC injections into the sciatic nerve resulted in the development of tumors within 2 months. **(B)** Representative immunohistochemical images of the endoneurial and epineurial compartments. Note the presence of widespread Ki67+ (red), Hoechst+ (blue) proliferative iSC (green) in the epineurial compartment compared to the endoneurial compartment. See inset (ˆˆ) for high-resolution example of localization (arrowheads). **(C)** iSCs injected into the dorsal column of the spinal cord formed tumors within 2 months. **(D)** Representative immunohistochemical images of the spinal cord. Note the presence of widespread Ki67+ (red), Hoechst+ (blue) proliferative iSC (green) in the spinal cord. See inset (^∗∗^) for high-resolution example of localization (arrowheads). **(E)** Quantification of the percentage of Ki67+ iSCs in the endoneurial compartment, epineurial compartment and spinal cord demonstrated a significant decrease in endoneurial compartment compared to other regions (One-way ANOVA, Tukey’s *posthoc* test, *n* = 6–8, **^∗^***p* < 0.01, **^∗∗^***p* < 0.001). **(F)** Representative immunocytochemical images of iSCs treated with conditioned media (CM). Note there are less Ki67+ (red) Hoechst+ (blue) iSCs in the cultures treated with endoneurial CM compared to epineurial, spinal cord or base media. **(G)** Quantification of the percentage of Ki67+ iSCs demonstrated a significant decrease in the percentage of Ki67+ iSCs under endoneurial CM conditions compared to all other groups (One-way ANOVA, Tukey’s *posthoc* test, *n* = 3, ^∗∗∗∗^*p* < 0.0001, ^∗∗∗^*p* < 0.001, ^∗∗^*p* < 0.01). Scale bars = **B** (50 μm), **E** (20 μm), **F** (50 μm).

**FIGURE 3:**
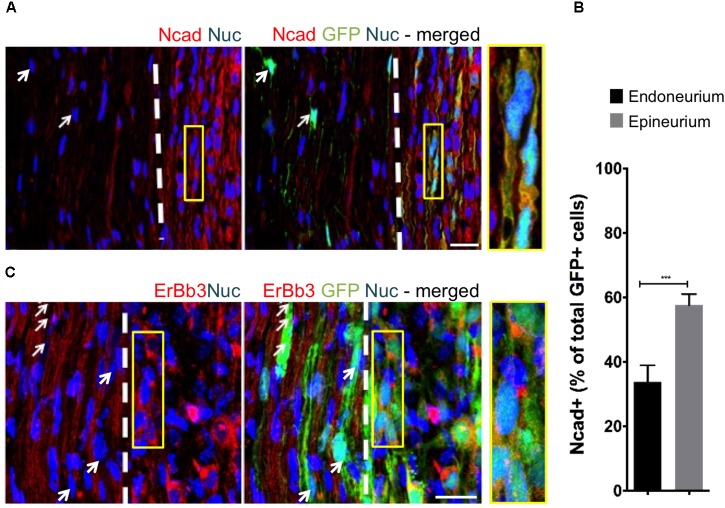
The fascicular microenvironment suppresses tumor-associated protein expression in MPNST. **(A)** Representative immunohistochemical images of the endoneurial and epineurial compartments. Note the presence of widespread Ncad+ (red), Hoechst+ (blue) iSC (green) in the epineurial compartment (inset) compared to the endoneurial compartment (arrow). See inset for high-resolution example of colocalization of proteins. **(B)** Quantification of the percentage of Ncad+ iSCs in endoneurial and epineurial compartments demonstrated a significant decrease in endoneurial compartment (Student’s *t*-test, *n* = 4, **^∗∗∗^***p* < 0.0008). **(C)** Representative immunohistochemical images of the endoneurial and epineurial compartment. Note the presence of widespread ErbB3+ (red), Hoechst+ (blue) iSC (green) in the epineurium (insert) compared to the endoneurium (arrows). See inset for high-resolution example of colocalization of proteins. Scale bars = **A** (20 μm), **C** (10 μm), Insets (10 μm, **A**; 5 μm, **C**).

To assess the effect of distinct nerve microenvironments on iSC tumor progression, GFP-labeled iSCs were injected into specific compartments (eg. endoneurium and epineurium) within the sciatic nerves of immune deficient rodents. Within 2 months post-transplant, in 88% of cases (8/9 animals), we observed the formation of tumors (**Figure 2A**). Importantly, there was a robust presence of Ki67+ GFP+ iSCs (Watanabe et al., 2001) and their location was strictly confined to the epineurial compartment (**Figure 2B**). Quantification of the percentage of Ki67+ GFP+ iSCs demonstrated a fourfold decrease in proliferation in the endoneurial compartment compared to the epineurial compartment (One-way ANOVA with Tukey’s *posthoc* test, *n* = 6, *p* < 0.001, **Figure 2E**). We also noted reduced expression of other cancer-associated proteins in iSCs within the endoneurial compartment compared to those within the epineurial compartment, including ErbB3 (Stonecypher et al., 2005) and N-cadherin (Flaiz et al., 2008), both associated with dysregulated growth factor signaling in tumor growth (Aplin et al., 1998; Hanahan and Weinberg, 2000) (**Figure 3**). We also performed identical injections of iSCs into the spinal cord. Similar to the epineurial compartment, highly proliferative and invasive iSC tumors formed in 88% of cases (9/10) (**Figures 2C–E**).

Finally, we asked whether secreted factors may be responsible for this context-dependent growth inhibition. To do this, we exposed iSCs to conditioned media from microdissected adult nerve endoneurium, epineurium or spinal cord tissues. Intriguingly, there was a ∼40% reduction in the percentage of proliferative iSCs when subjected to conditioned media from endoneurial tissue compared to media conditioned with epineurium or spinal cord tissue homogenates (One-way ANOVA, Tukey’s *posthoc* test, *n* = 3, endoneurium vs; depleted, *p* < 0.0001; spinal cord, *p* < 0.001; epineurium, *p* < 0.01, **Figures 2F,G**). Taken together, these findings suggest that endoneurial microenvironments are uniquely suppressive for Schwann cells predisposed for oncogenic growth.

### The Identification of Suppressive Factors in the Endoneurial Compartment

**FIGURE 4:**
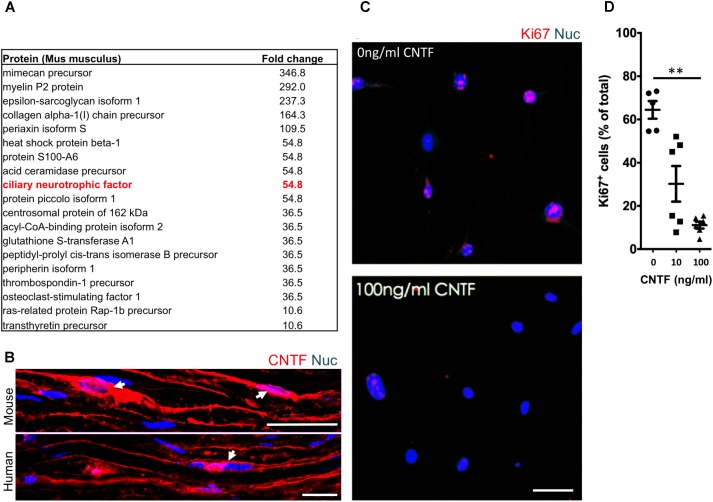
The identification of suppressive factors in the endoneurial compartment. **(A)** Fold change in candidate proteins (10–60 kDa) enriched in the endoneurium compared to spinal cord tissue. Ciliary neurotrophic factor (CNTF, red box), a growth factor known for its role in promoting differentiation of Schwann cells, was found at levels 5.5-fold higher in endoneurium tissue compared to spinal cord tissue. **(B)** Representative immunohistochemical images of uninjured mouse and human sciatic nerves demonstrating the presence of CNTF (red) in the cytoplasm of Schwann cells (blue). Note the presence of CNTF around the peri-nuclear area adjacent to nuclei (blue)—a cytoplasmic rich region of the myelinating Schwann cell. **(C)** Representative immunocytochemical images of iSCs (blue) treated with 0 ng/ml and 100 ng/ml CNTF. Note the reduction in Ki67+ (red) cells in CNTF treated conditions. **(D)** Quantification of the percentage of Ki67+ cells treated with 0, 10, or 100 ng/ml CNTF revealed a reduction in ki67+ cells at 100 ng/ml compared to 0 ng/ml (One-way ANOVA, Tukey’s *posthoc* test, *n* = 3, ^∗∗^*p* < 0.05). Scale bars = **B** (25 μm), **C** (50 μm).

To identify potential repressive signaling proteins (10–60 kDa) enriched in endoneurial tissue of the adult mouse sciatic nerve we employed tandem mass spectrometry to perform unbiased proteomic analysis. We found that CNTF—a growth factor known for promoting Schwann cell differentiation (Reynolds and Woolf, 1993), was 5.5-fold enriched in endoneurial tissue compared to spinal cord tissue (**Figure 4A**). We validated CNTF expression with immunohistochemistry on mouse and human adult uninjured nerves and found CNTF immunoreactivity restricted to cytoplasmic-rich regions of myelinating Schwann cells – most obviously at the perinuclear area (**Figure 4B**). Most importantly, we found that application of CNTF alone on iSCs *in vitro* caused a striking dose-dependent reduction in proliferation (**Figure 4C**). Quantification of this effect revealed up to a sixfold reduction in the percentage of Ki67+ iSCs when treated with 100ng/ml CNTF (One-way ANOVA, Tukey’s *posthoc* test, *n* = 3, ^∗∗^*p* < 0.05) (**Figure 4D**). Taken together, CNTF appears to be an important regulator of oncogenic Schwann cells and this data suggests that CNTF or small molecules targeting its downstream targets, may offer some therapeutic value toward controlling aberrant Schwann cells and MPNSTs.

## Discussion

These studies provide several major findings that has implications for MPNST and consequently peripheral nerve regeneration. First, we developed a reproducible *in vitro* model to study MPNST from isolated adult Schwann cells (termed iSCs) that following transplantation, share striking phenotypic resemblance to primary human MPNST tumors. Second, our results underscore the importance of regional microenvironments in promoting tumorigenic growth and identify the endoneurial compartment within the peripheral nerve as a unique microenvironment enriched in tumor suppressive factors. Third, by probing uniquely expressed proteins within the endoneurial compartment, we demonstrated an autonomous role for CNTF to block proliferation of iSCs mimicking the inhibition observed when grafted iSCs are contained within the endoneurial compartment *in vivo*. Together, these experiments underscore the plasticity of iSC, highlights their sensitivity to microenvironment, and identifies CNTF signaling as a potential therapeutic avenue for MPNST.

Therapeutic approaches that take advantage of the responsiveness of tumor forming cells to CNTF offers a promising approach to treat MPNST. First, tumor-forming cells remain responsive to this growth factor—which is already a potentially valuable finding given the large amount of pathogenic pathways present within cancer cells (Birindelli et al., 2001). Moreover, given the well-described role of CNTF-induced differentiation in healthy cells (Rathje et al., 2011; Johnson et al., 2014), we suspect that CNTF-induced cell cycle arrest in iSCs likely takes advantage of differentiation-induced cell cycle arrest, which has for a long time been a promising avenue to treat cancer (Nguyen et al., 2016). Like in MPNST, in normal health following nerve injury, proliferation of Schwann cells also occurs. But in contrast to the uncontrollable Schwann cell proliferation that occurs in MPNST, in normal health, these Schwann cells have the capacity to transition back into a mature differentiated myelinating state and contribute to regeneration. It is possible that CNTF-induced cell cycle arrest of iSCs piggy-backs on similar molecular pathways as regenerative-induced cell cycle arrest in normal health, but further study is needed.

Malignant peripheral nerve sheath tumors are mostly resistant to current standards of treatment (Ferner and Gutmann, 2002; Zehou et al., 2013), and surgical removal is often inadequate and debilitating as major nerves are sacrificed, given that the MPNST is often in close proximity to or in association with major nerves (Plotkin et al., 2013). At present, there are few early-stage diagnostic features that can adequately predict the risk of MPNST. In recent years, there have been several attempts to better understand the genetic bases of MPNST with the hope that this knowledge could be used for early detection, better predictions, and the discovery of new therapeutic targets. Recently, Rahrmann et al. (2013) used an animal model approach to investigate several likely genetic links (NF1, Pten, and EGFR) for PNSTs, finding that a subset of genes were key players in the formation of high-grade PNSTs (otherwise known as MPNST). Unfortunately, identified genes only partially accounted for the progression of benign tumors toward high-grade PNST formation and in most cases only when there was a combination of forced genetic mutations. Rodents generally formed either benign PNSTs, such as neurofibromas, or no tumors at all. Taken together, such findings suggest that, although there may be some genetic characteristics of MPNST, these characteristics do not autonomously dictate outcome. Rather, additional contributors, such as, epigenetic and environmental factors, must also play a role in MPNST development.

Our results support the notion that the cellular microenvironment plays a key role in predictability of tumor formation. When Ribiero and colleagues first made the seminal discovery that NF1-mutant Schwann cells *only* formed tumors post-injury, a logical conclusion for this phenomenon was an environmental cue associated with the injury must also play a role. Indeed there are several factors associated with injury (Gaudet et al., 2011) as well as associated with tumor progression (Brossier and Carroll, 2012) that overlap. Most researched, in relation to PNST, is the mast cell (Yang et al., 2008). But, in addition to mast cells, there are many other injury-associated factors that influence cancer cells. Regulatory T-cells thrive in the cancerous microenvironment, producing excess TGFβ1, preventing the proliferation of other immune cells reducing immune responses to tumors, as well as enhancing tumor cell proliferation (Franco et al., 2016). In addition, cancer associated fibroblasts (CAFs) have the ability to regulate malignant growth (Erez et al., 2010; Xing et al., 2010). Finally, another influencing factor for tumor progression is low pH (Jahde et al., 1982)— also known to be regulated in injury (Lengheden and Jansson, 1995). The fact that there is so much overlap with injury-associated factors and factors known to promote tumorigenesis, presupposes relationships between these factors and tumor progression (Brossier and Carroll, 2012). Interestingly, though Ribeiro et al. (2013) found that tumors did not form distal to the injury site – an area of the nerve that undergoes Wallerian degeneration post-injury and is, therefore, subject to similar injury cues, including the presence of mast cells and much of the factors described above (Gaudet et al., 2011). Such findings suggest that injury-associated factors are not the only mechanism at play in injury-associated Schwann cell-derived nerve sheath tumor progression. Consistent with Ribeiro et al. (2013), our results demonstrate that iSCs injected into the endoneurium of the distal stump, where injury cues are widespread, does not result in tumor formation. Together, such findings suggest that exposure to other factors at the injury site may be triggering malignant growth.

The fact that the perineurium is compromised when nerve injury occurs, resulting in long term dysfunction of the perineurium, including a chronic increase in permeability, *but* only at the injury site and not distally (Haftek and Thomas, 1968; Olsson and Kristensson, 1973) makes it plausible that a compromised nerve-barrier might contribute to Schwann cell-derived tumor formation and progression. Usually this specialized perineurial barrier prevents fibroblast-derived and adipocyte-derived factors, as well as large amounts of collagen in the epineurium (Sunderland, 1945; Riccardi, 2007; Weiss et al., 2016) to diffuse freely within the endoneurium where widespread Schwann cells are present (Norris et al., 1985; Verheijen et al., 2003). Given that several papers have shown that factors within the epineurial compartment can lead to tumor progression (Fang et al., 2014; Kuzet and Gaggioli, 2016; McDonald et al., 2016); and that factors within the endoneurial compartment reduce Schwann cell proliferation (Parrinello et al., 2008), including CNTF, it is not surprising that we have found that iSCs are highly tumorigenic in the epineurial compartment compared to the endoneurial compartment. Taken together, this dataset suggests that in order for tumor progression to occur, the growth-promoting factors within the epineurium override suppressive factors within the endoneurium, ultimately driving tumor formation in peripheral nerves.

Finally, our data offers a cautionary note regarding the use of adult Schwann cells for stem cell transplant-based therapies to treat nervous system injury and disease. Our work demonstrates that these cells can exhibit genomic instability and readily acquire aberrations following repeated *in vitro* expansion resulting in uncontrolled growth. As such, we strongly believe that adult Schwann cells, isolated from patients for subsequent autologous nervous system transplants, should undergo minimal *in vitro* processing, and be subjected to rigorous genetic screening before re-introduction into patients.

## Author Contributions

JAS, PA, NP, AM, RK, SS, ER, and JC contributed to execution of experiments. JAS, JB, RM, and WT designed the study and interpreted the data. JAS and JB prepared the manuscript.

## Conflict of Interest Statement

The authors declare that the research was conducted in the absence of any commercial or financial relationships that could be construed as a potential conflict of interest.
